# Environmental Impact of Diets Based on Established and Newly Proposed Dietary Guidelines: DASH, Mediterranean Diet and Dietary Guidelines for Americans 2025–2030-Style Diet

**DOI:** 10.3390/nu18091466

**Published:** 2026-05-03

**Authors:** Silvia García, Marina Ródenas-Munar, Cristina Bouzas, Josep A. Tur

**Affiliations:** Research Group on Community Nutrition and Oxidative Stress, University of the Balearic Islands-IUNICS, IDISBA & CIBEROBN (Physiopathology of Obesity and Nutrition CB12/03/30038), Guillem Colom Bldg, 07122 Palma de Mallorca, Spain; silvia.garcia@uib.es (S.G.); m.rodenas@uib.es (M.R.-M.);

**Keywords:** Mediterranean diet, DASH diet, Dietary Guidelines for Americans 2025–2030-style diet, sustainability, greenhouse gas emissions

## Abstract

Background: Dietary patterns face substantial environmental pressures, but few studies compare the ecological impact of those derived from widely implemented or recently proposed dietary guidelines. Updates to the American dietary pyramid have raised concerns about these environmental implications. Objectives: To compare greenhouse gas emissions (GHGE), water use, land use, and energy use across three dietary patterns: Dietary Approaches to Stop Hypertension (DASH), Mediterranean diet (MD), and Dietary Guidelines for Americans (DGA) 2025–2030-style diet. Design: Comparative analysis of modeled seven-day diets based on each guideline. Methods: Representative diets were constructed following food-based recommendations for each pattern. Environmental indicators for all food items were obtained from the Agribalyse^®^ 3.0.1 database. Daily GHGE, water use, land use, and energy use were calculated. Differences between patterns were assessed using one-way ANOVA with Bonferroni corrections, before and after adjustment for total dietary energy intake. Results: The DGA 2025–2030-style diet showed significantly higher GHGE and energy use compared with DASH and MD (*p* < 0.001), while no significant differences were observed between DASH and MD. No significant differences were found for water or land use across dietary patterns, which may be due to the heterogeneous contribution of individual food items to these indicators across dietary patterns. The DGA 2025–2030-style diet also had the highest total energy intake (*p* < 0.001). After adjustment for dietary energy intake, only GHGE differences remained significant (F = 6.187, *p* = 0.009), with the DGA 2025–2030-style diet showing the highest values and the MD the lowest. Conclusions: Dietary guidelines should integrate environmental sustainability criteria alongside nutritional recommendations. Reducing the environmental impact of diets may be achieved by promoting dietary patterns such as the MD and DASH diets, and by limiting high-impact foods characteristic of the DGA 2025–2030-style diet, particularly those contributing to higher GHGE. These strategies could support the transition toward diets that are both nutritionally adequate and environmentally sustainable.

## 1. Introduction

Food systems are currently recognized as one of the major contributors to environmental degradation, accounting for a substantial release of global greenhouse gas emissions (GHGE), water use, land occupation and deterioration, and energy demand [1,2,3,4]. Recent estimates indicate that food systems contribute between approximately one-quarter and one-third of total anthropogenic GHGE, while also accounting for around 70% of global freshwater withdrawals and exerting major pressures on land use and biodiversity across the supply chain [5,6,7,8]. In addition, food loss and waste contribute approximately 8–10% of global GHGE, further exacerbating environmental impacts [9,10]. In this context, dietary patterns have emerged as a key modifiable factor linking human health and environmental sustainability. As a result, increasing attention has been paid to the environmental impact of dietary recommendations promoted through national and international food-based dietary guidelines [11,12]. Importantly, dietary choices can be understood as the result of both voluntary individual behaviors and adherence to structured dietary guidelines, each associated with distinct environmental footprints. While voluntary dietary shifts (e.g., reducing meat consumption) can directly lower environmental impacts, institutional dietary recommendations play a critical role in shaping population-level consumption patterns and, consequently, large-scale environmental outcomes.

Several dietary patterns have been widely studied not only for their health benefits but also for their potential environmental implications [13,14,15,16,17]. Among them, the Mediterranean diet (MD) has been well-defined as an environmentally sustainable model [18,19,20,21,22], and the Dietary Approaches to Stop Hypertension (DASH) diet [23] has also started to be studied in the same context [24]. Both diets are often cited as examples of healthy dietary models, characterized by a high consumption of plant-based foods and a limited intake of red and processed meats [25,26,27,28,29,30]. In contrast, dietary guidelines based on more Westernized food models have been associated with higher environmental pressures and worse health outcomes [13,31,32,33], although their sustainability implications remain a subject of debate due to recent changes in their dietary recommendations.

Recent studies have further explored the environmental impacts of different dietary patterns, consistently showing that plant-rich diets tend to generate lower GHGE and resource use compared with animal-based dietary patterns, although variability exists depending on specific food choices and methodological approaches (e.g., life cycle assessment frameworks) [34]. Despite these advances, comparative analyses based on standardized and guideline-derived dietary models remain limited.

Lately, the release of the new American dietary pyramid in an inverted format has generated considerable public and scientific discussion [35]. This “inverted” representation refers to a graphical reorganization of traditional dietary pyramids [36], where protein-rich foods (including both animal- and plant-based sources) are placed at the base to emphasize higher relative consumption, while other food groups such as grains occupy a reduced proportion in the overall structure. Critics have raised concerns regarding both its nutritional messaging and its potential environmental consequences [37,38]. However, empirical evidence evaluating the environmental impact of dietary patterns derived from these recommendations remains scarce. This gap highlights the need for comparative analyses that translate dietary guidelines into realistic dietary patterns and assess their environmental footprints using standardized indicators. Environmental impact assessments of diets most commonly focus on indicators such as carbon dioxide (CO_2_) emissions, water use, land use, and energy use, as these parameters are among the most extensively studied and consistently reported in the scientific literature. Together, they provide a comprehensive overview of the pressures exerted by dietary choices on climate change, natural resources, and energy systems [39,40,41,42,43].

Therefore, the aim of this study is to compare the environmental impact of three dietary patterns based on different dietary recommendations: the DASH diet, the MD, and the new Dietary Guidelines for Americans (DGA) 2025–2030-style dietary pattern.

The selection of the DASH diet, the MD, and the DGA 2025–2030 dietary pattern is based on their global relevance and contrasting characteristics. While the MD and DASH diets are widely recognized as evidence-based healthy dietary models with increasing international adoption, the DGA 2025–2030-style diet reflects a commonly followed Western dietary pattern and incorporates recent updates from DGA 2025–2030, making it particularly relevant for evaluating emerging trends and their environmental implications.

Therefore, the aim of this study is not only to compare the environmental impact of these three dietary patterns but also to identify potential leverage points for reducing their environmental footprint, thereby contributing to the development of more sustainable dietary recommendations.

## 2. Methods

### 2.1. Design

Three dietary pattern-based diets were defined to reflect dietary recommendations from distinct nutritional models: the DASH diet [23], the MD [44], and the DGA 2025–2030-style diet [35].

Each dietary pattern was translated into a seven-day representative menu designed to reflect real-life adherence to the respective dietary guidelines (DASH, MD, and DGA 2025–2030-style diet), following approaches commonly used in previous dietary modeling studies based on multi-day menus [45,46]. Rather than using identical food items across diets, food selection was guided by the specific recommendations of each dietary pattern, including emphasized and limited food groups, target servings, and overall dietary structure.

A standardized framework was applied to ensure comparability in terms of meal structure and food group coverage (similar number of meals per day and inclusion of all major food groups). However, diets were not designed to be isocaloric, as the primary aim was to reflect realistic dietary patterns based on guideline recommendations. This non-isocaloric design was intentionally adopted to reflect real-world implementation of dietary guidelines, as these recommendations do not prescribe identical energy intakes but rather define food group distributions and portion ranges. Constructing isocaloric diets would require artificial modifications that could distort the intrinsic structure and relative composition of each dietary pattern. To address potential confounding due to differences in total energy intake, environmental indicators were additionally analyzed after adjustment for dietary energy, which is a commonly used approach in dietary environmental impact studies. This dual analytical strategy allows both the assessment of absolute environmental burdens and the evaluation of diet composition independent of caloric intake.

Within this framework, individual food items were selected based on their relevance within each dietary pattern, their frequency of recommendation in the corresponding guidelines, and their representativeness of commonly consumed foods in real-world settings. Differences in food items across dietary patterns therefore reflect inherent distinctions between the guidelines (higher inclusion of whole grains and low-fat dairy in DASH, greater emphasis on plant-based fats and fish in the MD, and higher emphasis on protein-rich foods, moderate-to-high dairy intake, and a relatively lower proportion of grains in the DGA 2025–2030-style diet), rather than arbitrary choices [23,35,44]. All menus were developed by trained dietitians to ensure nutritional plausibility and alignment with guideline recommendations. Final daily diets were quantified in grams per day for each food item and aggregated by food group for analysis. Full detailed menus, including all food items, quantities (g/day), and nutritional composition (total energy and macronutrient content), are provided in Supplementary Table S1 to ensure full reproducibility of the dietary patterns.

### 2.2. GHGs, Energy, Water, and Land Use Calculations

Environmental impact estimates were derived from the Agribalyse^®^ 3.0.1 database, developed by the French Agency for Ecological Transition (ADEME) and linked to the CIQUAL French food composition table [47]. Agribalyse^®^ integrates life cycle inventory data for agricultural and food products and is complemented by Ecoinvent^®^ v.1, which supplies information on non-agricultural processes such as electricity production, transportation, and imported goods. Together, these databases are designed to represent production systems and market conditions typical of European countries. The database is structured according to life cycle assessment principles and provides reference environmental impact values for food products expressed per kilogram of product. The assessment includes primary production, processing, packaging, transportation, distribution, retail, consumer preparation, and packaging disposal. Transport from retail to households and food waste at the consumer level are not considered. Thus, the system boundaries follow a cradle-to-retail approach, including all stages from raw material production to consumer preparation, but excluding household food waste and post-retail transport. A detailed description of the database methodology has been published elsewhere [48].

For the present analysis, four environmental indicators were selected due to their frequent use and relevance in the scientific literature: GHGE, water use, energy use, and land use. GHGE were expressed as kilograms of carbon dioxide equivalents (kg CO_2_-eq). Water use was quantified in cubic meters (m^3^), accounting for regional water scarcity and depletion. Energy use was expressed in megajoules (MJ) and reflected the consumption of non-renewable energy sources, including fossil fuels and uranium. Land use, which is closely linked to biodiversity impacts, was assessed using the ecosystem quality (Pt) indicator, representing deviations from a natural reference state; higher point values indicate greater land-related biodiversity degradation. These four selected environmental parameters were calculated for each one of the dietary pattern-based diets separately.

The functional unit was defined as one daily diet within each 7-day dietary pattern. Environmental impacts were calculated for each day and expressed as mean daily values averaged across the 7-day cycle.

### 2.3. Comparison Between Diets

Comparisons between dietary patterns were performed by assessing total daily GHGE, water use, energy use and land use. Absolute values for each environmental indicator were reported across dietary patterns to highlight differences in environmental burdens associated with the three dietary models. Subsequent comparisons of mean values were conducted as the primary analytical approach.

In addition to environmental indicators, total daily energy intake (kcal/day) was calculated for each diet, facilitating interpretation of environmental outcomes in the context of overall dietary composition. This variable was used for descriptive purposes and, when appropriate, for adjustment in statistical analyses. Macronutrient composition (including proteins, carbohydrates, and fats) was also calculated to ensure nutritional plausibility and comparability between dietary patterns (see Supplementary Table S1). The contribution of individual food items was also quantified in absolute amounts (g/day), including the total weight per diet, and was analyzed for each day within the three seven-day dietary plans (detailed versions of the diets are provided in Supplementary Table S1).

### 2.4. Statistics

Descriptive statistics were used to summarize the environmental indicators associated with each dietary pattern. Continuous variables were expressed as absolute daily values for each dietary pattern-based diet. Given that the diets were constructed from predefined dietary patterns rather than derived from population-based observations, the analysis focused primarily on descriptive comparisons between dietary patterns.

One-way analysis of variance (ANOVA) was conducted to evaluate differences in environmental indicators and dietary energy among the three dietary patterns. When significant differences were detected, Bonferroni post hoc tests were applied to identify pairwise differences between diets. Spearman correlation analyses were also conducted to explore associations between environmental indicators (GHGE, energy use, water use, and land use) within each dietary pattern across the seven modeled days. Given the small sample size per group and the non-parametric nature of the data, Spearman’s rank correlation coefficient was used. Correlation coefficients (ρ) and corresponding significance levels were reported, with statistical significance set at *p* < 0.05.

A second set of analyses was performed after adjusting environmental indicators for dietary energy intake to account for differences in caloric content across diets. The unadjusted ANOVA models were considered the primary analyses, while energy-adjusted models were performed as sensitivity analyses to assess the robustness of the results to differences in total dietary energy intake. Statistical analyses were conducted using SPSS statistical software version 27.0 (SPSS Inc., Chicago, IL, USA). Statistical significance was set at *p* < 0.05. Additionally, a post hoc power analysis was conducted using G*Power software v.1 based on the observed effect sizes (η^2^ = 0.177–0.219; corresponding effect size f = 0.46–0.53). The analysis was performed using an alpha level of 0.05, a total sample size of 21 observations (7 days per dietary pattern), and 3 groups. Effect sizes were derived from the raw (unadjusted) ANOVA models. The estimated statistical power ranged between 0.39 and 0.50. These estimates should be interpreted in the context of modeled dietary data rather than independent population samples.

## 3. Results

Table 1 shows the absolute environmental impact values of the three dietary patterns (DASH, MD, and DGA 2025–2030-style diet) across the seven analyzed days. Overall, the DGA 2025–2030-style diet showed higher values for GHGE, energy use, and total dietary energy intake than DASH and MD. Higher day-to-day variability was observed for land use and water use, particularly in the MD, which showed the widest range of values across the seven days.

The one-way ANOVA results comparing the environmental indicators across the three dietary patterns are shown in Table 2. Significant differences between dietary patterns were observed for GHGE, energy use, and total dietary energy intake. GHGE differed significantly among diets (F = 10.815, *p* < 0.001, η^2^ = 0.177). Post hoc Bonferroni comparisons show that the DGA 2025–2030-style diet presented significantly higher GHGE than both DASH and MD, whereas no significant differences were observed between DASH and MD. Similarly, energy use differed significantly across dietary patterns (F = 12.454, *p* < 0.001, η^2^ = 0.192). The DGA 2025–2030-style diet showed significantly higher energy use compared with both DASH and MD, whereas no significant differences were observed between these two patterns.

Significant differences were also found for dietary energy intake (F = 13.624, *p* < 0.001, η^2^ = 0.219), with the DGA 2025–2030-style diet showing the highest caloric intake, followed by the MD and DASH diet. In contrast, no statistically significant differences were observed for land use (*p* = 0.383) or water use (*p* = 0.383) among the three dietary patterns.

Spearman correlation analyses were conducted within each dietary pattern to assess the relationships between environmental indicators across the seven modeled days (Table 3). In the DASH diet, a strong positive correlation was observed between GHGE and energy use (ρ = 0.750), while a strong inverse relationship was found between land use and energy use (ρ = −0.893, *p* < 0.01). Correlations involving water use were generally weak and inconsistent. In the MD, GHGE showed a perfect positive correlation with energy use (ρ = 1.000, *p* < 0.01), and both GHGE and energy use were positively correlated with water use (ρ = 0.786, *p* < 0.05). In the DGA 2025–2030-style diet, GHGE and energy use were also perfectly correlated (ρ = 1.000, *p* < 0.01), while correlations with land and water use were generally weak to moderate and less consistent across indicators. Overall, GHGE and energy use showed the most consistent and strongest associations across all dietary patterns, whereas land and water use exhibited more heterogeneous and diet-dependent relationships.

After adjusting environmental indicators for dietary energy intake, differences between dietary patterns remained significant only for GHGE (Table 4). The ANOVA showed significant differences among diets (F = 6.187, *p* = 0.009, η^2^ = 0.407). Post hoc Bonferroni comparisons show that the DGA 2025–2030-style diet had significantly higher GHGE than MD, while the DASH diet showed intermediate values and did not differ significantly from the other two dietary patterns. No differences were found for land use (*p* = 0.670), water use (*p* = 0.225), or energy use (*p* = 0.068) after adjusting for dietary energy intake.

## 4. Discussion

The current study compared the environmental impact of three dietary patterns derived from established and recently proposed dietary recommendations: the DASH diet, the MD, and the DGA 2025–2030-style diet. Current results show that the DGA 2025–2030-style diet exhibited the highest environmental burden, particularly in terms of GHGE and energy use, whereas the DASH and MD patterns demonstrated substantially lower impacts. After adjusting for total dietary energy intake, differences in GHGE persisted, with the DGA 2025–2030-style diet remaining the most environmentally intensive and the MD showing the lowest values. In contrast, no differences were observed across dietary patterns for water use or land use. Given the exploratory nature of the energy-adjusted analyses, results were interpreted as secondary and used to assess consistency of findings rather than as independent confirmatory tests. These findings highlight the relevance of dietary composition, beyond caloric intake, in shaping environmental outcomes, and underscore the need to evaluate the sustainability implications of emerging dietary guidelines.

Beyond total dietary energy intake, the environmental differences observed across dietary patterns are largely explained by their underlying food composition. Although energy adjustment improves comparability between diets, it does not account for the heterogeneous environmental intensity of different food groups. Foods of animal origin, particularly ruminant meat and dairy products, are consistently associated with higher GHGE and energy use due to enteric fermentation, feed production, and intensive supply chains, whereas most plant-based foods show substantially lower emissions per unit of energy provided [6,49,50]. The persistence of significant GHGE differences after energy adjustment in this study indicates that the environmental burden of the DGA 2025–2030-style diet is driven not only by caloric quantity, but also by the intrinsic environmental intensity of the foods emphasized within this dietary pattern. Accordingly, the representative food groups and compositional features underlying each dietary pattern, and their contribution to the observed environmental impacts, are described and discussed in the following paragraphs.

The DASH diet was originally developed to reduce blood pressure and cardiovascular risk. It emphasizes foods naturally rich in potassium, calcium, magnesium, fiber, and lean protein, while limiting saturated fats, added sugars, and highly processed foods. Core recommended foods include fruits, vegetables, whole grains, legumes, nuts, low-fat dairy, and lean meats, whereas fatty meats, full-fat dairy, tropical oils, and processed foods are discouraged. Daily serving recommendations for a 2.000 kcal diet typically include 6–8 servings of grains, 4–5 servings each of fruits and vegetables, 2–3 servings of low-fat dairy, and ≤6 servings of lean meats, poultry, or fish, with sodium intake capped at 2300 mg/day [23,29,51]. This plant-forward structure, combined with moderate animal protein intake, aligns the DASH diet with patterns known to have lower environmental footprints. Previous research has shown that diets rich in plant foods and low in red and processed meats tend to reduce GHGE and energy use [49,52].

The DGA 2025–2030-style diet modeled in this study reflects the recently proposed inverted American dietary pyramid, which places a stronger emphasis on protein intake, approximately 1.2–1.6 g/kg body weight/day, sourced from both animal and plant foods. It also prioritizes dairy (unsweetened), fruits, vegetables, and healthy fats, while grains occupy a smaller proportion of total intake. Recommended daily servings include approximately three servings of protein foods, three servings of vegetables, two servings of fruit, and 2–4 servings of grains, with fats contributing around 10% of total energy intake. These guidelines derive from the most recent DGA (2025–2030), which promotes nutrient-dense foods but still includes substantial amounts of animal-based products [35]. Although nutritionally oriented toward improving metabolic health, this pattern retains several features characteristic of Western diets, higher intake of animal proteins, dairy, and energy-dense foods, which are known contributors to elevated GHGE and resource use.

The Mediterranean diet is characterized by high consumption of plant-based foods, fruits, vegetables, legumes, whole grains, nuts, and olive oil as the primary fat source. It includes moderate intake of dairy, fish, and poultry, and low consumption of red and processed meats. This dietary pattern prioritizes complex carbohydrates, unsaturated fats, and lean protein sources, and has been consistently associated with both health benefits and reduced environmental impact [28,53,54]. Its emphasis on plant foods and limited reliance on ruminant meat aligns with sustainability principles [21,55,56], although certain Mediterranean staples (e.g., nuts, olive oil) can have high water or land-use demands depending on production methods [57,58,59].

Our results further highlight the role of food-type “hotspots” in shaping environmental indicators, particularly land use and water use. While plant-forward diets generally reduce GHGE, specific plant-based foods such as nuts, olive oil, and some fruits and vegetables can exhibit high water or land requirements depending on irrigation intensity, regional water scarcity, and agricultural practices [57,60,61]. This helps explain the substantial intra-pattern variability observed within the MD and the lack of statistically significant differences in land and water use across dietary patterns. These findings underscore that plant-based diets are not uniformly low-impact across all environmental dimensions and reinforce the need for multidimensional sustainability assessments. The use of a seven-day modeled diet may also contribute to intra-pattern variability, as day-to-day fluctuations in food choices can influence environmental indicators.

Taken together, our diet comparison results underscore the central role of the food type, rather than energy intake alone, in determining environmental impact. These results are consistent with previous research showing that dietary patterns rich in plant-based foods and lower in red and processed meats tend to have substantially reduced carbon footprints and energy demands. The high GHGE and energy use associated with the DGA 2025–2030-style diet likely reflect its higher proportion of animal-based foods and energy-dense items, which are known to be environmentally intensive. Even though the new American pyramid aims to improve nutritional quality, its environmental implications remain concerning.

These findings point to an inherent trade-off between nutritional optimality and environmental sustainability. Foods that are central to healthy dietary patterns, such as dairy products, fish, nuts, and vegetable oils, may carry relatively high environmental footprints, despite their established health benefits. Therefore, environmentally sustainable diets should not be conceptualized as nutritionally restrictive, but rather as dietary patterns that balance health outcomes with environmental constraints [3]. From a theoretical perspective, this challenges binary classifications of diets as either “healthy” or “sustainable” and instead supports integrated frameworks that jointly optimize both dimensions. From a managerial and policy perspective, these results suggest that sustainable dietary guidelines should prioritize food-type substitutions, portion sizes, and diversification of protein sources rather than caloric intake alone. Shifting consumption away from ruminant meat toward plant-based proteins, moderating dairy intake, and favoring minimally processed foods may offer meaningful reductions in environmental impact while maintaining nutritional adequacy and safety. Such strategies are consistent with emerging global frameworks, including the Planetary Health Diet, which aim to align human health with planetary boundaries [3,4].

Our results reinforce the need to integrate sustainability considerations into dietary guidelines. While nutritional adequacy remains essential, the growing contribution of food systems to global environmental change demands that public health recommendations account for environmental impacts. In this context, the Planetary Health Diet proposed by the EAT-Lancet Commission provides an illustrative framework that integrates nutritional quality with environmental limits, emphasizing predominantly plant-based dietary patterns as a strategy to support both human and planetary health [3]. While this framework is widely recognized as a reference for aligning dietary health and environmental sustainability, it has also been subject to discussion regarding its cost, cultural acceptability, and feasibility across different socio-economic and regional contexts. Despite these limitations, it remains a key reference framework for operationalizing sustainability criteria and applying them to other dietary patterns, including those assessed in this study. Our findings highlight the comparatively favorable environmental profiles of the MD and DASH diets, emerging as promising models for sustainable healthy eating. Moreover, our findings underscore the need to incorporate environmental considerations into DGA 2025–2030, where sustainability is not currently addressed despite the clear environmental burden observed in the DGA 2025–2030-style diet. Incorporating environmental criteria into future dietary guidelines could support both human and planetary health.

### Strengths and Limitations

This study has several strengths, including the use of standardized life cycle assessment data from Agribalyse^®^, the construction of realistic dietary models based on established guidelines, and the inclusion of energy-adjusted analyses. The non-isocaloric nature of the modeled diets represents a potential source of variability, as differences in total energy intake may influence absolute environmental impact estimates. However, this approach was chosen to preserve the structural integrity of each dietary pattern as defined by existing guidelines. Energy-adjusted analyses were therefore conducted to isolate the effect of dietary composition, showing that key differences, particularly in GHGE, persisted after adjustment. A major strength of this study is also the comprehensive assessment of the four environmental indicators most frequently used and recognized in scientific literature (GHGE, land use, water use, and energy use). However, limitations must also be acknowledged. First, the dietary patterns analyzed represent hypothetical diets derived from guidelines rather than actual population intakes. Although the estimated statistical power was moderate, it should be interpreted with caution, because the data were derived from modeled dietary scenarios rather than independent population samples. Therefore, the analysis reflects structured variability within dietary patterns rather than stochastic variability across individuals. Second, although Agribalyse^®^ is one of the most comprehensive life cycle assessment databases in Europe, environmental impacts may vary across countries and production systems. In particular, the use of a European-based database to model a DGA 2025–2030-style dietary pattern may not fully capture differences in agricultural practices, supply chains, transportation systems, and food sourcing specific to the United States. These differences could influence the absolute environmental impact estimates and may lead to some degree of over- or underestimation of the environmental footprint of the DGA 2025–2030-style diet. However, it is important to emphasize that the primary objective of this study was to compare dietary patterns rather than to reproduce country-specific food systems. Therefore, the use of a consistent life cycle assessment framework across all dietary models ensures internal comparability, allowing observed differences to be mainly attributed to dietary composition rather than to geographic variability in production systems. Furthermore, key environmental drivers, particularly GHGE, are largely determined by food type (animal- versus plant-based products) [62,63], suggesting that the relative ranking of dietary patterns is unlikely to be substantially altered by regional differences in production systems. Nevertheless, future research could incorporate region-specific databases or sensitivity analyses to further assess the robustness of these findings. Finally, only four environmental indicators were selected; additional parameters such as biodiversity loss, eutrophication, or food waste could further refine sustainability evaluations.

## 5. Conclusions

This study suggests that the DGA 2025–2030 dietary pattern may be associated with a higher environmental burden, particularly in terms of GHGE and energy use, even after adjusting for caloric intake. In contrast, the MD and DASH diets appear to represent more environmentally sustainable alternatives within the context of the modeled scenarios.

However, these findings are based on hypothetical seven-day menus derived from dietary guidelines and do not necessarily reflect real-world population-level dietary patterns. Therefore, the results should be interpreted with caution. Nevertheless, the study highlights the potential value of incorporating sustainability considerations into dietary guidelines and supports the promotion of dietary models that may benefit both human and planetary health.

## Figures and Tables

**Table 1:** Absolute environmental impact values of three dietary patterns.

	DASH	MD	DGA 2025–2030-Style Diet
DAY 1			
GHGE (CO_2_eq)	2.97	2.04	4.31
Land use (PT)	143.03	98.75	149.00
Water use (M3)	5.45	5.75	6.18
Energy use (MJ)	33.87	27.00	50.41
Energy amount (kcal)	1953.7	1861.5	1903.1
DAY 2			
GHGE (CO_2_eq)	2.74	2.91	4.40
Land use (PT)	216.42	194.28	131.28
Water use (M3)	5.36	5.18	3.31
Energy use (MJ)	29.68	33.36	53.46
Energy amount (kcal)	1576.3	1648.4	2269.6
DAY 3			
GHGE (CO_2_eq)	3.24	4.11	7.59
Land use (PT)	89.49	717.52	357.29
Water use (M3)	7.10	7.10	7.64
Energy use (MJ)	42.71	52.65	57.06
Energy amount (kcal)	1844.3	1607.6	1898.8
DAY 4			
GHGE (CO_2_eq)	2.57	2.81	4.38
Land use (PT)	141.80	134.35	159.84
Water use (M3)	6.43	6.82	4.36
Energy use (MJ)	34.96	31.99	50.70
Energy amount (kcal)	1534.8	1786.7	2204.1
DAY 5			
GHGE (CO_2_eq)	2.95	3.43	8.17
Land use (PT)	105.80	150.49	418.72
Water use (M3)	9.52	7.61	6.36
Energy use (MJ)	37.93	40.01	61.40
Energy amount (kcal)	1506.5	1792.5	2250.0
DAY 6			
GHGE (CO_2_eq)	2.42	2.37	4.18
Land use (PT)	164.71	158.27	249.54
Water use (M3)	8.77	4.27	5.63
Energy use (MJ)	26.22	28.53	45.77
Energy amount (kcal)	1587.0	1806.6	1967.4
DAY 7			
GHGE (CO_2_eq)	2.51	4.47	9.11
Land use (PT)	190.01	145.28	325.04
Water use (M3)	7.53	25.58	5.03
Energy use (MJ)	30.23	52.88	74.88
Energy amount (kcal)	1432.2	1809.8	1934.9

Abbreviations: CO_2_eq: Carbon dioxide equivalents; DASH: Dietary Approaches to Stop Hypertension; DGA: Dietary Guidelines for Americans; GHGE: Greenhouse Gas Emissions; MD: Mediterranean Diet.

**Table 2:** One-factor ANOVA and Bonferroni post hoc between environmental parameters, diet energy and three dietary patterns.

	DASH	MD	DGA 2025–2030-Style Diet	df	F	η^2^	*p*-Value
	Mean (SD)—[CI 95%]						
GHGE (CO_2_eq)	2.77 (0.29) ^a^ [2.49–3.04]	3.16 (0.89) ^c^ [2.33–3.98]	6.02 (2.17) ^ac^ [4.01–8.03]	2, 18	10.815	0.177	<0.001
Land use (PT)	150.18 (44.64) [108.88–191.47]	228.42 (217.54) [27.22–429.61]	255.82 (113.86) [150.50–361.12]	2, 18	1.013	0.000	0.383
Water use (M3)	7.17 (1.58) [5.70–8.62]	8.90 (7.44) [2.01–15.78]	5.50 (1.42) [4.18–6.81]	2, 18	1.012	0.000	0.383
Energy use (MJ)	33.66 (5.55) ^a^ [28.52–38.79]	38.06 (10.86) ^c^ [28.01–48.10]	56.24 (9.63) ^ac^ [47.33–65.14]	2, 18	12.454	0.192	<0.001
Energy (kcal)	1633.55 (190.94) ^a^ [1456.95–1810.14]	1759.05 (93.44) ^c^ [1672.63–1845.46]	2061.14 (171.08) ^ac^ [1902.91–2219.37]	2, 18	13.624	0.219	<0.001

Abbreviations: CO_2_eq: Carbon dioxide equivalents; DASH: Dietary Approaches to Stop Hypertension; DGA: Dietary Guidelines for Americans; df: degrees of freedom; F: Fisher value; GHGE: Greenhouse Gas Emissions; MD: Mediterranean Diet; η^2^: Eta squared. Superscript letters indicate significant pairwise differences between dietary patterns according to Bonferroni post hoc tests (*p* < 0.05): a = DASH vs. DGA 2025–2030-style diet; b = DASH vs. MD; c = MD vs. DGA 2025–2030-style diet.

**Table 3:** Spearman correlation coefficients between crude environmental indicators within each dietary pattern.

	GHGE (ρ)	Land Use (ρ)	Water Use (ρ)	Energy Use (ρ)
DASH				
GHGE (ρ)	1	−0.607	−0.286	0.750
Land use (ρ)	−0.607	1	−0.357	−0.893 **
Water use (ρ)	−0.286	−0.357	1	0.143
Energy use (ρ)	0.750	−0.893 **	0.143	1
DM				
GHGE (ρ)	1	0.429	0.786 *	1.000 **
Land use (ρ)	0.429	1	−0.143	0.429
Water use (ρ)	0.786 *	−0.143	1	0.786 *
Energy use (ρ)	1.000 **	0.429	0.786 *	1
US				
GHGE (ρ)	1	0.571	0.143	1.000 **
Land use (ρ)	0.571	1	0.714	0.571
Water use (ρ)	0.143	0.714	1	0.143
Energy use (ρ)	1.000 **	0.571	0.143	1

* *p* < 0.05; ** *p* < 0.01; ρ: correlation coefficient.

**Table 4:** One-factor ANOVA and Bonferroni post hoc between environmental parameters, three dietary patterns, adjusted by diet energy.

	DASH	MD	DGA 2025–2030-Style Diet	df	F	η^2^	*p*-Value
	Mean (SD)—[CI 95%]						
GHGE (CO_2_eq)	3.06 (0.27) [2.81–3.31] ^a^	3.2 (0.99) [2.34–4.17] ^c^	5.31 (2.04) [3.41–7.20] ^a c^	2, 18	6.187	0.407	0.009
Land use (PT)	169.22 (59.39) [114.29–224.15]	242.80 (249.57) [11.98–473.62]	225.70 (101.34) [131.97–319.42]	2, 18	0.409	0.043	0.670
Water use (M3)	8.05 (2.27) [5.95–10.16]	9.05 (7.3) [2.26–15.84]	4.88 (1.49) [3.49–6.27]	2, 18	1.623	0.153	0.225
Energy use (MJ)	37.26 (5.82) [31.87–42.64]	39.27 (12.34) [27.85–50.68]	49.46 (10.04) [40.17–58.74]	2, 18	3.129	0.258	0.068

Abbreviations: CO_2_eq: Carbon dioxide equivalents; DASH: Dietary Approaches to Stop Hypertension; DGA: Dietary Guidelines for Americans; df: degrees of freedom; F: Fisher value; GHGE: Greenhouse Gas Emissions; MD: Mediterranean Diet; η^2^: Eta squared. Superscript letters indicate significant pairwise differences between dietary patterns according to Bonferroni post-hoc tests (*p* < 0.05): a = DASH vs. DGA 2025–2030-style diet; b = DASH vs. MD; c = MD vs. DGA 2025–2030-style diet.

## Data Availability

The original contributions presented in this study are included in the article/Supplementary Materials. Further inquiries can be directed to the corresponding author.

## Supplementary Materials

The following supporting information can be downloaded at: https://www.mdpi.com/article/10.3390/nu18091466/s1, Supplementary Table S1. A seven-day representative menu designed to reflect real-life adherence to the respective dietary guidelines (DASH, MD, DGA 2025–2030-style diet).
