# Supplementary material for: Environmental Impact of Diets Based on Established and Newly Proposed Dietary Guidelines: DASH, Mediterranean Diet and Dietary Guidelines for Americans 2025–2030-Style Diet
Source: Nutrients. 2026 May 3;18(9):1466. doi: 10.3390/nu18091466 (PMC13164914; doi:10.3390/nu18091466)
Supplement: Supplementary file 1 [file nutrients-18-01466-s001.zip › nutrients-4268967-supplementary.pdf]

The DM and DASH data presented here have been obtained from the BEDCA database (https://www.bedca.net/bdpub/). and the data for the U.S. diet have been obtained from the USDA database (https://fdc.nal.usda.gov/). In the U.S. section. foods marked with an asterisk (*) indicate that no information was available in the USDA database. and data from BEDCA were used instead.

Table S1. A seven-day representative menu designed to reflect real-life adherence to the respective dietary guidelines (DASH, MD, DGA 2025–2030-style diet).

DAY 1

			Calories (kcal)		Proteins (g)		Fats (g)		Carbohydrates (g)		Observations
**Diet**	**Food type**	**Weight (grams)**	**100g**	**Kcal diet**	**100g**	**Grams diet**	**100g**	**Grams diet**	**100g**	**Grams diet**	
**DASH_b**	Whole wheat bread	80	251	200.8	10.9	8.72	3	2.4	44	35.2	
**DASH_b**	Tomato	50	19	9.5	0.9	0.45	0.1	0.05	3.5	1.75	
**DASH_b**	Extra virgin olive oil	10	888	88.8	0	0	100	10	0	0	
**DASH_b**	Unsalted fresh cheese	75	198	148.5	2.5	1.875	15.4	11.55	2.5	1.875	
**DASH_b**	Mandarin orange	150	40	60	0.8	1.2	0	0	9	13.5	
**DASH_b**	Herbal tea	200	0	0	0	0	0	0	0	0	
**DASH_mm**	Low-fat plain yogurt	125	46	57.5	4.3	5.375	0.32	0.4	6.3	7.875	
**DASH_mm**	Walnuts	20	595	119	14	2.8	63.28	12.656	3.3	0.66	
**DASH_mm**	Banana	200	89	178	1.2	2.4	0.3	0.6	20	40	
**DASH_l**	Baked salmon	150	182	273	18.4	27.6	12	18	0	0	
**DASH_l**	Broccoli	100	26	26	3	3	0.4	0.4	2.4	2.4	
**DASH_l**	Brown rice	60	386	231.6	7.5	4.5	2.6	1.56	81.3	48.78	
**DASH_l**	Kiwi	100	52	52	1.1	1.1	0.5	0.5	10.6	10.6	
**DASH_ma**	Hummus	40	360	144	8.9	3.56	2.5	1	18.7	7.48	
**DASH_ma**	Carrot	20	34	6.8	0.8	0.16	0.3	0.06	7	1.4	
**DASH_d**	Grilled chicken	125	166	207.5	20.85	26.0625	9.25	11.5625	0	0	
**DASH_d**	Spinach	150	22	33	2.7	4.05	0.8	1.2	1	1.5	
**DASH_d**	Whole wheat bread	20	251	50.2	10.9	2.18	3	0.6	44	8.8	

**DASH_d**	Pear	150	45	67.5	0.4	0.6	0	0	10.6	15.9	
**Total**				**1953.7**		**95.6325**		**72.5385**		**197.72**	
**EEUU_b**	Scrambled eggs	120	143	171.6	12.3	14.76	10.3	12.36	0.9	1.08	
**EEUU_b**	Whole wheat bread	80	254	203.2	12.3	9.84	3.55	2.84	43.1	34.48	
**EEUU_b**	Avocado	80	223	178.4	1.81	1.448	20.3	16.24	8.32	6.656	
**EEUU_b**	Tomato	50	22	11	0.7	0.35	0.42	0.21	3.24	1.62	
**EEUU_b**	Coffee with milk*	200	38	76	1.7	3.4	2.36	4.72	2.45	4.9	
**EEUU_mm**	Apple	200	65	130	0.15	0.3	0.15	0.3	15.6	31.2	
**EEUU_mm**	Almonds	20	626	125.2	21.4	4.28	51.1	10.22	20	4	
**EEUU_l**	Baked chicken	200	127	254	21.4	42.8	4.78	9.56	0	0	
**EEUU_l**	Green beans	150	40	60	1.97	2.955	0.28	0.42	7.41	11.115	
**EEUU_l**	Quinoa*	70	306	214.2	13.8	9.66	5.56	3.892	49.2	34.44	
**EEUU_ma**	Greek yogurt	150	59	88.5	10.3	15.45	0.37	0.555	3.64	5.46	
**EEUU_d**	Hake*	200	65	130	11.93	23.86	1.8	3.6	0	0	
**EEUU_d**	Broccoli	150	39	58.5	2.57	3.855	0.34	0.51	6.27	9.405	
**EEUU_d**	Cauliflower	100	28	28	1.64	1.64	0.24	0.24	4.72	4.72	
**EEUU_d**	Baked potatoes	150	73	109.5	1.81	2.715	0.26	0.39	16	24	
**EEUU_d**	Kiwi	100	65	65	1.01	1.01	0.64	0.64	13.8	13.8	
**Total**				**1903.1**		**138.323**		**66.697**		**186.876**	
**MD_b**	Whole wheat bread	80	251	200.8	10.9	8.72	3	2.4	44	35.2	
**MD_b**	Tomato	50	19	9.5	0.9	0.45	0.1	0.05	3.5	1.75	
**MD_b**	Extra virgin olive oil	10	888	88.8	0	0	100	10	0	0	
**MD_b**	Turkey breast	50	158	79	20.18	10.09	8.5	4.25	0	0	
**MD_b**	Coffee with milk	200	38	76	1.7	3.4	2.36	4.72	2.45	4.9	
**MD_b**	Orange	200	38	76	0.8	1.6	0	0	8.6	17.2	
**MD_mm**	Plain yogurt (fortified)	125	64	80	3.2	4	3.9	4.875	3.9	4.875	
**MD_mm**	Walnuts	20	595	119	14	2.8	63.28	12.656	3.3	0.66	
**MD_l**	Lentils	40	310	124	24.76	9.904	1.17	0.468	48.69	19.476	
**MD_l**	Rice	40	387	154.8	7	2.8	0.9	0.36	86	34.4	
**MD_l**	Onion	25	26	6.5	1.25	0.3125	0	0	5.3	1.325	
**MD_l**	Tomato	25	19	4.75	0.9	0.225	0.1	0.025	3.5	0.875	

**MD_l**	Carrot	50	34	17	0.8	0.4	0.3	0.15	7	3.5	
**MD_l**	Swiss chard	60	21	12.6	2.1	1.26	0.2	0.12	2.7	1.62	
**MD_l**	Extra virgin olive oil	10	888	88.8	0	0	100	10	0	0	
**MD_l**	Banana	200	89	178	1.2	2.4	0.3	0.6	20	40	
**MD_ma**	Apple	200	50	100	0.3	0.6	0	0	12	24	
**MD_d**	Sardines	200	140	280	18.1	36.2	7.5	15	0	0	
**MD_d**	Boiled potato	150	73	109.5	2.2	3.3	0.2	0.3	15.2	22.8	
**MD_d**	Zucchini	150	17	25.5	1.8	2.7	0.2	0.3	2	3	
**MD_d**	Strawberries	100	36	36	0.7	0.7	0.5	0.5	7	7	
**Total**				**1866.55**		**91.8615**		**66.774**		**222.581**	

DAY 2

			**Calories (kcal)**		**Proteins (g)**		**Fats (g)**		**Carbohydrates (g)**		**Observations**
**Diet**	**Food type**	**Weight (grams)**	**100g**	**Kcal diet**	**100g**	**Grams diet**	**100g**	**Grams diet**	**100g**	**Grams diet**	
**DASH_b**	Whole wheat bread	80	251	200.8	10.9	8.72	3	2.4	44	35.2	
**DASH_b**	Tomato	50	19	9.5	0.9	0.45	0.1	0.05	3.5	1.75	
**DASH_b**	Extra virgin olive oil	10	888	88.8	0	0	100	10	0	0	
**DASH_b**	Unsalted fresh cheese (Burgos cheese)	75	198	148.5	2.5	1.875	15.4	11.55	2.5	1.875	
**DASH_b**	Apricot	150	42	63	0.8	1.2	0	0	9.5	14.25	
**DASH_b**	Herbal tea	200	0	0	0	0	0	0	0	0	
**DASH_mm**	Low-fat plain yogurt	125	46	57.5	4.3	5.375	0.32	0.4	6.3	7.875	
**DASH_mm**	Walnuts	20	595	119	14	2.8	63.28	12.656	3.3	0.66	
**DASH_mm**	Strawberries	100	36	36	0.7	0.7	0.5	0.5	7	7	
**DASH_l**	Grilled chicken	125	166	207.5	20.85	26.0625	9.25	11.5625	0	0	

**DASH_l**	Tomato	75	19	14.25	0.9	0.675	0.1	0.075	3.5	2.625	
**DASH_l**	Cucumber	75	12	9	0.7	0.525	0.1	0.075	2	1.5	
**DASH_l**	Unsalted fresh cheese	30	198	59.4	2.5	0.75	15.4	4.62	2.5	0.75	
**DASH_i**	Avocado	70	137	95.9	1.5	1.05	12	8.4	0.4	0.28	
**DASH_I**	Banana	200	89	178	1.2	2.4	0.3	0.6	20	40	
**DASH_ma**	Carrot	20	34	6.8	0.8	0.16	0.3	0.06	7	1.4	
**DASH_ma**	Hummus (boiled chickpeas)	40	360	144	8.9	3.56	2.5	1	18.7	7.48	
**DASH_d**	Egg	100	150	150	12.5	12.5	11.1	11.1	0	0	
**DASH_d**	Broccoli	75	26	19.5	3	2.25	0.4	0.3	2.4	1.8	
**DASH_d**	Cauliflower	75	18	13.5	2	1.5	0.175	0.13125	2.1	1.575	
**DASH_d**	Potato	150	73	109.5	2.2	3.3	0.2	0.3	15.2	22.8	
**DASH_d**	Pear	150	45	67.5	0.4	0.6	0	0	10.6	15.9	
	**Total**			**1498.85**		**76.4525**		**75.77975**		**164.72**	
**EEUU_b**	Greek yogurt	150	59	88.5	10.3	15.45	0.37	0.555	3.64	5.46	
**EEUU_b**	Blueberries	100	64	64	0.7	0.7	0.31	0.31	14.6	14.6	
**EEUU_b**	Oats	60	382	229.2	13.5	8.1	5.89	3.534	68.7	41.22	
**EEUU_b**	Coffee with milk*	200	38	76	1.7	3.4	2.36	4.72	2.45	4.9	
**EEUU_mm**	Whole wheat bread	60	254	152.4	12.3	7.38	3.55	2.13	43.1	25.86	
**EEUU_mm**	Hummus	40	243	97.2	7.35	2.94	17.1	6.84	14.9	5.96	
**EEUU_mm**	Cottage cheese	50	103	51.5	11.6	5.8	4.22	2.11	4.6	2.3	
**EEUU_l**	Walnuts	20	730	146	14.6	2.92	69.7	13.94	10.9	2.18	
**EEUU_l**	Chicken	125	127	158.75	21.4	26.75	4.78	5.975	0	0	
**EEUU_l**	Brown rice	60	366	219.6	7.25	4.35	3.31	1.986	76.7	46.02	
**EEUU_l**	Carrot	50	48	24	0.94	0.47	0.35	0.175	10.3	5.15	
**EEUU_l**	Onion	50	36	18	0.89	0.445	0.13	0.065	7.68	3.84	
**EEUU_l**	Tomato	50	22	11	0.7	0.35	0.42	0.21	3.24	1.62	
**EEUU_ma**	Pear	150	64	96	0.38	0.57	0.16	0.24	15.1	22.65	
**EEUU_d**	Cod	200	66	132	16.1	32.2	1.48	2.96	0	0	

**EEUU_d**	Arugula	50	31	15.5	1.65	0.825	0.32	0.16	5.37	2.685	
**EEUU_d**	Tomato	75	22	16.5	0.7	0.525	0.42	0.315	3.24	2.43	
**EEUU_d**	Avocado	50	223	111.5	1.81	0.905	20.3	10.15	8.32	4.16	
**Total**				**1707.65**		**114.08**		**56.375**		**191.035**	
**MD_b**	Whole wheat bread	80	251	200.8	10.9	8.72	3	2.4	44	35.2	2 slices
**MD_b**	Ripe tomato	50	19	9.5	0.9	0.45	0.1	0.05	3.5	1.75	Crushed or sliced
**MD_b**	Extra virgin olive oil	10	888	88.8	0	0	100	10	0	0	1 tablespoon
**MD_b**	Serrano ham	30	319	95.7	28.8	8.64	22.6	6.78	0.2	0.06	2 thin slices
**MD_b**	Coffee with semi-skimmed milk	200	38	76	1.7	3.4	2.37	4.74	2.45	4.9	1 cup
**MD_mm**	Hazelnuts	20	656	131.2	12	2.4	56.25	11.25	10.5	2.1	1 handful
**MD_mm**	Apple	150	52	78	0.3	0.45	0	0	12	18	1 medium piece
**MD_l**	Whole wheat pasta (dry)	70	347	242.9	13.4	9.38	2.5	1.75	66.2	46.34	1 normal plate
**MD_l**	Chicken breast	125	105	131.25	23.1	28.875	1.2	1.5	0	0	grilled or sautéed
**MD_l**	Zucchini	150	17	25.5	1.8	2.7	0.2	0.3	2	3	sautéed with pasta
**MD_l**	Onion	50	26	13	1.25	0.625	0	0	5.3	2.65	sautéed with pasta
**MD_l**	Olive oil	10	888	88.8	0	0	100	10	0	0	1 tablespoon
**MD_l**	Orange	200	38	76	0.8	1.6	0	0	8.6	17.2	1 big unit
**MD_ma**	Fresh cheese (cow's milk)	80	198	158.4	2.5	2	15.4	12.32	2.5	2	1 individual portion
**MD_d**	Green beans	150	28	42	2.2	3.3	0.4	0.6	3.7	5.55	1 plate of boiled vegetables
**MD_d**	Carrot	50	34	17	0.8	0.4	0.3	0.15	7	3.5	boiled or raw (salat)
**MD_d**	Boiled chicken egg	60	150	90	12.5	7.5	11.1	6.66	0	0	1 unit (soft-boiled or scrammbled)
**MD_d**	Olive oil (for dressing)	10	888	88.8	0	0	100	10	0	0	1 tablespoon
**MD_d**	Whole natural yogurt	125	64	80	3.2	4	3.9	4.875	3.9	4.875	1 unit
**MD_d**	Strawberries	150	36	54	0.7	1.05	0.5	0.75	7	10.5	1 cup of strawberries
**Total**				**1733.65**		**85.49**		**84.125**		**157.625**	

DAY 3

			Calories (kcal)		Proteins (g)		Fats (g)		Carbohydrates (g)		Observations
**Diet**	**Food type**	**Weight (grams)**	**100g**	**Kcal diet**	**100g**	**Grams diet**	**100g**	**Grams diet**	**100g**	**Grams diet**	
**DASH_b**	Low-fat plain yogurt	125	46	57.5	4.3	5.375	0.32	0.4	6.3	7.875	
**DASH_b**	Oats	50	401	200.5	16.9	8.45	6.9	3.45	66.3	33.15	
**DASH_b**	Banana	200	89	178	1.2	2.4	0.3	0.6	20	40	
**DASH_b**	Herbal tea	200	0	0	0	0	0	0	0	0	
**DASH_mm**	Whole wheat bread	80	251	200.8	10.9	8.72	3	2.4	44	35.2	
**DASH_mm**	Avocado	80	137	109.6	1.5	1.2	12	9.6	0.4	0.32	
**DASH_mm**	Tomato	50	19	9.5	0.9	0.45	0.1	0.05	3.5	1.75	
**DASH_mm**	Kiwi	100	52	52	1.1	1.1	0.5	0.5	10.6	10.6	
**DASH_I**	Whole wheat pasta	80	347	277.6	13.4	10.72	2.5	2	66.2	52.96	
**DASH_l**	Eggplant	50	20	10	0.7	0.35	0.2	0.1	3.8	1.9	
**DASH_l**	Red bell pepper	50	29	14.5	1.3	0.65	0.6	0.3	4.5	2.25	
**DASH_l**	Onion	50	26	13	1.25	0.625	0	0	5.3	2.65	
**DASH_l**	Extra virgin olive oil	10	888	88.8	0	0	100	10	0	0	
**DASH_l**	Pear	150	45	67.5	0.4	0.6	0	0	10.6	15.9	
**DASH_ma**	Almonds	20	589	117.8	19.13	3.826	45.22	9.044	6.2	1.24	
**DASH_ma**	Orange	200	38	76	0.8	1.6	0	0	8.6	17.2	
**DASH_d**	Zucchini	150	17	25.5	1.8	2.7	0.2	0.3	2	3	
**DASH_d**	Boiled potato	50	73	36.5	2.2	1.1	0.2	0.1	15.2	7.6	

**DASH_d**	Fresh cod	150	83	124.5	18.2	27.3	1	1.5	0	0	
**DASH_d**	Sweet potato	150	101	151.5	1.2	1.8	0.3	0.45	23	34.5	
**Total**				**1753.6**		**78.966**		**40.794**		**268.095**	
**EEUU_b**	Whole wheat bread	80	251	200.8	12.3	9.84	3.55	2.84	43.1	34.48	
**EEUU_b**	Eggs	120	143	171.6	245.388	12.3	17.589	10.3	14.729	0.9	
**EEUU_b**	Fresh spinach	100	27	27	2.85	2.85	0.62	0.62	2.41	2.41	
**EEUU_b**	Tomato	50	22	11	0.7	0.35	0.42	0.21	3.24	1.62	
**EEUU_b**	Coffee with milk*	200	38	76	1.7	3.4	2.36	4.72	2.45	4.9	
**EEUU_mm**	Banana	200	98	196	0.74	1.48	0.29	0.58	23	46	
**EEUU_mm**	Walnuts	20	730	146	14.6	2.92	69.7	13.94	10.9	2.18	
**EEUU_l**	Salmon	200	197	394	20.3	40.6	13.1	26.2	0	0	
**EEUU_l**	Carrots	75	48	36	0.94	0.705	0.35	0.2625	10.3	7.725	
**EEUU_l**	Asparagus	75	28	21	1.44	1.08	0.22	0.165	5.1	3.825	
**EEUU_ma**	Greek yogurt	150	59	88.5	10.3	15.45	0.37	0.555	3.64	5.46	
**EEUU_ma**	Strawberries	100	36	36	0.64	0.64	0.22	0.22	7.96	7.96	
**EEUU_d**	Beef*	200	110	220	20.19	40.38	3.06	6.12	0	0	
**EEUU_d**	Red bell pepper	75	31	23.25	0.9	0.675	0.13	0.0975	6.65	4.9875	
**EEUU_d**	Onion	50	36	18	0.89	0.445	0.13	0.065	7.68	3.84	
**EEUU_d**	Eggplant	75	26	19.5	0.85	0.6375	0.12	0.09	5.4	4.05	
**EEUU_d**	Quinoa	70	306	214.2		0		0		0	
**Total**				**1898.85**		**133.7525**		**66.985**		**130.3375**	
**MD_b**	Whole wheat bread	60	251	150.6	10.9	6.54	3	1.8	44	26.4	2-3 slices
**MD_b**	Extra virgin olive oil	10	888	88.8	0	0	100	10	0	0	1 tablespoon
**MD_b**	Fresh cheese (cow's milk)	80	198	158.4	2.5	2	15.4	12.32	2.5	2	1 individual portion
**MD_b**	Semi-skimmed milk	200	45	90	3.2	6.4	1.6	3.2	4.6	9.2	1 cup
**MD_b**	Plums (without skin)	150	48	72	0.6	0.9	0	0	11	16.5	2-3 medium pieces

**MD_mm**	Walnuts	20	595	119	14	2.8	63.28	12.656	3.3	0.66	1 handful
**MD_mm**	Whole natural yogurt	125	64	80	3.2	4	3.9	4.875	3.9	4.875	1 unit
**MD_l**	Chickpeas (dry)	70	336	235.2	19.3	13.51	6.3	4.41	49.25	34.475	1 normal plate
**MD_l**	Fresh spinach	150	22	33	2.7	4.05	0.8	1.2	1	1.5	1 plate of boiled vegetables
**MD_l**	Fresh cod	125	83	103.75	18.2	22.75	1	1.25	0	0	1 individual steak
**MD_l**	Olive oil (for cooking/dressing)	10	888	88.8	0	0	100	10	0	0	1 tablespoon
**MD_l**	Pear	150	45	67.5	0.4	0.6	0	0	10.6	15.9	1 unit
**MD_ma**	Mandarin orange	150	40	60	0.8	1.2	0	0	9	13.5	1 medium piece
**MD_d**	Boiled potato	150	73	109.5	2.2	3.3	0.2	0.3	15.2	22.8	1 big potato
**MD_d**	Boiled chicken egg	60	150	90	12.5	7.5	11.1	6.66	0	0	2 medium units
**MD_d**	Lettuce	100	16	16	1.125	1.125	0.6	0.6	1.4	1.4	salad base
**MD_d**	Cucumber	50	12	6	0.7	0.35	0.1	0.05	2	1	part of the salad
**MD_d**	Olive oil (for dressing)	10	888	88.8	0	0	100	10	0	0	1 tablespoon
**Total**				**1657.35**		**77.025**		**79.321**		**150.21**	

DAY 4

			**Calories (kcal)**		**Proteins (g)**		**Fats (g)**		**Carbohydrates (g)**		**Observations**
**Diet**	**Food type**	**Weight (grams)**	**100g**	**Kcal diet**	**100g**	**Grams diet**	**100g**	**Grams diet**	**100g**	**Grams diet**	
**DASH_b**	Low-fat plain yogurt	125	46	57.5	4.3	5.375	0.32	0.4	6.3	7.875	

**DASH_b**	Oats	50	401	200.5	16.9	8.45	6.9	3.45	66.3	33.15	
**DASH_b**	Kiwi	100	52	52	1.1	1.1	0.5	0.5	10.6	10.6	
**DASH_b**	Herbal tea	200	0	0	0	0	0	0	0	0	
**DASH_b**	Whole wheat bread	80	251	200.8	10.9	8.72	3	2.4	44	35.2	
**DASH_b**	Avocado	80	223	178.4		0		0		0	
**DASH_mm**	Tomato	50	19	9.5	0.9	0.45	0.1	0.05	3.5	1.75	
**DASH_mm**	Chickpeas (dry)	60	336	201.6	19.3	11.58	6.3	3.78	49.25	29.55	
**DASH_mm**	Red bell pepper	50	29	14.5	1.3	0.65	0.6	0.3	4.5	2.25	
**DASH_l**	Onion	50	26	13	1.25	0.625	0	0	5.3	2.65	
**DASH_l**	Tomato	50	19	9.5	0.9	0.45	0.1	0.05	3.5	1.75	
**DASH_l**	Mandarin orange	150	40	60	0.8	1.2	0	0	9	13.5	
**DASH_ma**	Almonds	20	589	117.8	19.13	3.826	45.22	9.044	6.2	1.24	
**DASH_ma**	Banana	200	89	178	1.2	2.4	0.3	0.6	20	40	
**DASH_d**	Spinach	100	22	22	2.7	2.7	0.8	0.8	1	1	
**DASH_d**	Tomato sauce	40	19	7.6	0.9	0.36	0.1	0.04	3.5	1.4	
**DASH_d**	Onion	50	26	13	1.25	0.625	0	0	5.3	2.65	
**DASH_d**	Whole wheat bread	20	251	50.2	10.9	2.18	3	0.6	44	8.8	
**DASH_d**	Apricot	150	42	63	0.8	1.2	0	0	9.5	14.25	
**Total**				**1391.4**		**51.891**		**22.014**		**207.615**	
**EEUU_b**	Whole wheat bread	80	254	203.2	12.3	9.84	3.55	2.84	43.1	34.48	
**EEUU_b**	Avocado	80	223	178.4	1.81	1.448	20.3	16.24	8.32	6.656	
**EEUU_b**	Chicken egg	60	143	85.8	122.694	12.3	17.589	10.3	14.729	0.9	
**EEUU_b**	Coffee with milk*	200	38	76	1.7	3.4	2.36	4.72	2.45	4.9	
**EEUU_mm**	Greek yogurt	150	59	88.5	10.3	15.45	0.37	0.555	3.64	5.46	
**EEUU_mm**	Banana	200	98	196	0.74	1.48	0.29	0.58	23	46	
**EEUU_mm**	Corn flakes*	50	381	190.5	6	3	3	1.5	3.4	1.7	
**EEUU_l**	Pork ribs*	200	279	558	17.1	34.2	23.6	47.2	0	0	
**EEUU_l**	Potato	150	73	109.5	1.81	2.715	0.26	0.39	16	24	
**EEUU_l**	Broccoli	150	39	58.5	2.57	3.855	0.34	0.51	6.27	9.405	
**EEUU_ma**	Hummus	60	243	145.8	7.35	4.41	17.1	10.26	14.9	8.94	
**EEUU_ma**	Carrot	30	48	14.4	0.94	0.282	0.35	0.105	10.3	3.09	
**EEUU_d**	Hake*	200	65	130	11.93	23.86	1.8	3.6	0	0	

**EEUU_d**	Sweet potato	150	78	117	1.58	2.37	0.38	0.57	17.3	25.95	
**EEUU_d**	Peas	150	80	120	4.73	7.095	1.15	1.725	12.7	19.05	
**Total**				**2271.6**		**125.705**		**101.095**		**190.531**	
**MD_b**	Whole wheat bread	80	251	200.8	10.9	8.72	3	2.4	44	35.2	2 large slices
**MD_b**	Extra virgin olive oil	10	888	88.8	0	0	100	10	0	0	1 tablespoon
**MD_b**	Ripe tomato	100	19	19	0.9	0.9	0.1	0.1	3.5	3.5	1 medium piece
**MD_b**	Semi-skimmed milk	200	45	90	3.2	6.4	1.6	3.2	4.6	9.2	1 cup
**MD_b**	Orange	200	38	76	0.8	1.6	0	0	8.6	17.2	1 large unit
**MD_mm**	Almonds	25	589	147.25	19.13	4.7825	45.22	11.305	6.2	1.55	1 handful
**MD_mm**	Whole natural yogurt	125	64	80	3.2	4	3.9	4.875	3.9	4.875	1 unit
**MD_l**	Chickpeas (dry)	70	336	235.2	19.3	13.51	6.3	4.41	49.25	34.475	1 regular plate
**MD_l**	Fresh Swiss chard	150	21	31.5	2.1	3.15	0.2	0.3	2.7	4.05	1 plate of cooked vegetables
**MD_l**	Fresh tuna	125	119	148.75	22	27.5	3.3	4.125	0	0	1 individual fillet
**MD_l**	Extra virgin olive oil	10	888	88.8	0	0	100	10	0	0	1 tablespoon
**MD_l**	Peach	150	39	58.5	0.6	0.9	0	0	9	13.5	1 medium piece
**MD_ma**	Fresh cheese (cow’s milk)	80	193	154.4	2.5	2	15.4	12.32	2.5	2	1 individual portion
**MD_d**	Quinoa (dry)	50	306	153	13.8	6.9	5.56	2.78	49.2	24.6	Half a cup (dry)
**MD_d**	Red bell pepper	100	29	29	1.3	1.3	0.6	0.6	4.5	4.5	In the quinoa salad
**MD_d**	Onion	50	26	13	1.25	0.625	0	0	5.3	2.65	In the quinoa salad
**MD_d**	Chicken egg	60	150	90	12.5	7.5	11.1	6.66	0	0	1 large cooked unit
**MD_d**	Extra virgin olive oil	10	888	88.8	0	0	100	10	**0**	0	1 tablespoon

MD_d	Apple	150	50	75	0.3	0.45	0	0	12	18	1 medium piece
Total				1792.8		90.2375		83.075		175.3	

DAY 5

			Calories (kcal)		Proteins (g)		Fats (g)		Carbohydrates (g)		Observations
Diet	Food type	Weight (grams)	100g	Kcal diet	100g	Grams diet	100g	Grams diet	100g	Grams diet	
DASH_b	Whole wheat bread	80	251	200.8	10.9	8.72	3	2.4	44	35.2	
DASH_b	Hummus	40	360	144	8.9	3.56	2.5	1	18.7	7.48	
DASH_b	Tomato	50	19	9.5	0.9	0.45	0.1	0.05	3.5	1.75	
DASH_b	Mandarin orange	150	40	60	0.8	1.2	0	0	9	13.5	
DASH_b	Herbal tea	200	0	0	0	0	0	0	0	0	
DASH_b	Low-fat plain yogurt	125	46	57.5	4.3	5.375	0.32	0.4	6.3	7.875	
DASH_mm	Almonds	20	589	117.8	19.13	3.826	45.22	9.044	6.2	1.24	
DASH_mm	Banana	200	89	178	1.2	2.4	0.3	0.6	20	40	
DASH_mm	Hake	150	65	97.5	11.93	17.895	1.8	2.7	0	0	
DASH_l	Peas	50	62	31	5.4	2.7	0.5	0.25	8.8	4.4	
DASH_l	Green beans	50	28	14	2.2	1.1	0.4	0.2	3.7	1.85	
DASH_l	White asparagus	50	24	12	1.9	0.95	0.3	0.15	3.4	1.7	
DASH_l	Boiled potato	150	73	109.5	2.2	3.3	0.2	0.3	15.2	22.8	
DASH_l	Orange	200	38	76	0.8	1.6	0	0	8.6	17.2	
DASH_ma	Whole wheat bread	80	251	200.8	10.9	8.72	3	2.4	44	35.2	
DASH_ma	Avocado	80	137	109.6	1.5	1.2	12	9.6	0.4	0.32	
DASH_ma	Tomato	50	19	9.5	0.9	0.45	0.1	0.05	3.5	1.75	
DASH_d	Turkey breast	50	107	53.5	24.12	12.06	0.99	0.495	0	0	
DASH_d	Zucchini	50	17	8.5	1.8	0.9	0.2	0.1	2	1	
DASH_d	Tomato	50	19	9.5	0.9	0.45	0.1	0.05	3.5	1.75	
DASH_d	Onion	50	26	13	1.25	0.625	0	0	5.3	2.65	
DASH_d	Brown rice	60	386	231.6	7.5	4.5	2.6	1.56	81.3	48.78	
DASH_d	Kiwi	100	52	52	1.1	1.1	0.5	0.5	10.6	10.6	

**Total**				**1381.3**		**83.081**		**31.849**		**257.045**	
**EEUU_b**	Large chicken egg	120	143	171.6	245.388	12.3	17.589	10.3	14.729	0.9	2 units
**EEUU_b**	Smoked salmon*	50	181	90.5	25.4	12.7	8.7	4.35	0	0	
**EEUU_b**	Whole wheat bread	40	247	98.8	12.3	4.92	3.55	1.42	43.1	17.24	1 slices
**EEUU_b**	Avocado	80	223	178.4	1.81	1.448	20.3	16.24	8.32	6.656	1/2 unit
**EEUU_b**	Coffee with milk*	200	38	76	1.7	3.4	2.36	4.72	2.45	4.9	1 cup
**EEUU_mm**	Greek yogurt (plain. low-fat)	150	59	88.5	10.3	15.45	0.37	0.555	3.64	5.46	
**EEUU_mm**	Almonds	20	626	125.2	21.4	4.28	51.1	10.22	20	4	1 handful
**EEUU_l**	Chicken thigh (roasted)	200	188	376	17.1	34.2	13.4	26.8	0	0	
**EEUU_l**	Broccoli (cooked)	150	39	58.5	2.57	3.855	0.34	0.51	6.27	9.405	1 portion of vegetables
**EEUU_l**	Cauliflower (cooked)	100	28	28	1.64	1.64	0.24	0.24	4.72	4.72	1/2 portion of vegetables
**EEUU_l**	Olive oil	15	888	133.2	0	0	100	15	0	0	1.5 spoons
**EEUU_l**	Brown rice (cooked)	80	366	292.8	7.25	5.8	3.31	2.648	76.7	61.36	small portion
**EEUU_l**	Kiwi	100	65	65	1.01	1.01	0.64	0.64	13.8	13.8	1 portion
**EEUU_ma**	Cottage cheese	50	103	51.5	11.6	5.8	4.22	2.11	4.6	2.3	1 individual portion
**EEUU_d**	Lean beef (steak)*	200	184	368	110	404.8	20.19	74.2992	3.06	11.2608	0
**EEUU_d**	Asparagus	150	28	42	1.44	2.16	0.22	0.33	5.1	7.65	1 portion of vegetables
**EEUU_d**	Green beans	100	40	40	1.97	1.97	0.28	0.28	7.41	7.41	1/2 portion of vegetables
**EEUU_d**	Olive oil	15	888	133.2	0	0	100	15	0	0	1.5 spoons
**EEUU_d**	Apple	150	65	97.5	0.15	0.225	0.15	0.225	15.6	23.4	1 portion of fruit
**Total**				**2514.7**		**515.958**		**185.8872**		**180.4618**	
**MD_b**	Whole wheat bread	60	251	150.6	10.9	6.54	3	1.8	44	26.4	2 medium slices
**MD_b**	Extra virgin olive oil	10	888	88.8	0	0	100	10	0	0	1 tablespoon
**MD_b**	Avocado	50	137	68.5	1.5	0.75	12	6	0.4	0.2	half small piece
**MD_b**	Semi-skimmed milk	200	45	90	3.2	6.4	1.6	3.2	4.6	9.2	1 cup

**MD_b**	Orange	200	38	76	0.8	1.6	0	0	8.6	17.2	1 big unit
**MD_mm**	Hazelnuts	20	656	131.2	12	2.4	56.25	11.25	10.5	2.1	1 handful
**MD_mm**	Whole natural yogurt	125	64	80	3.2	4	3.9	4.875	3.9	4.875	1 unit
**MD_l**	Brown rice (dry)	70	386	270.2	7.5	5.25	2.6	1.82	81.3	56.91	1 normal plate
**MD_l**	Chicken breast (grilled)	125	105	131.25	23.1	28.875	1.2	1.5	0	0	steamed
**MD_l**	Zucchini	100	17	17	1.8	1.8	0.2	0.2	2	2	sautéed
**MD_l**	Leek	30	24	7.2	1.6	0.48	0.3	0.09	3.7	1.11	sautéed
**MD_l**	Extra virgin olive oil	10	888	88.8	0	0	100	10	0	0	1 tablespoon
**MD_l**	Banana	120	89	106.8	1.2	1.44	0.3	0.36	20	24	1 medium piece
**MD_ma**	Apple	150	50	75	0.3	0.45	0	0	12	18	1 medium piece
**MD_d**	Green beans	150	28	42	2.2	3.3	0.4	0.6	3.7	5.55	1 plate of vegetables
**MD_d**	Fresh hake	150	65	97.5	11.93	17.895	1.8	2.7	0	0	1 big steak
**MD_d**	Extra virgin olive oil	10	888	88.8	0	0	100	10	0	0	1 tablespoon
**MD_d**	Whole natural yogurt	125	64	80	3.2	4	3.9	4.875	3.9	4.875	1 unit
**MD_d**	Strawberries	150	36	54	0.7	1.05	0.5	0.75	7	10.5	1 cup
**Total**				**1743.65**		**86.23**		**70.02**		**182.92**	

DAY 6

			**Calories (kcal)**		**Proteins (g)**		**Fats (g)**		**Carbohydrates (g)**		**Observations**
**Diet**	**Food type**	**Weight (grams)**	**100g**	**Kcal diet**	**100g**	**Grams diet**	**100g**	**Grams diet**	**100g**	**Grams diet**	
**DASH_b**	Low-fat plain yogurt	125	46	57.5	4.3	5.375	0.32	0.4	6.3	7.875	
**DASH_b**	Strawberries	100	36	36	0.7	0.7	0.5	0.5	7	7	
**DASH_b**	Herbal tea	200	0	0	0	0	0	0	0	0	

**DASH_mm**	Whole wheat bread	80	251	200.8	10.9	8.72	3	2.4	44	35.2	
**DASH_mm**	Extra virgin olive oil	10	888	88.8	0	0	100	10	0	0	
**DASH_mm**	Unsalted fresh cheese	75	198	148.5	2.5	1.875	15.4	11.55	2.5	1.875	
**DASH_mm**	Tomato	50	19	9.5	0.9	0.45	0.1	0.05	3.5	1.75	
**DASH_l**	Brown rice	70	386	270.2	7.5	5.25	2.6	1.82	81.3	56.91	
**DASH_l**	Broccoli	50	26	13	3	1.5	0.4	0.2	2.4	1.2	
**DASH_l**	Carrot	50	34	17	0.8	0.4	0.3	0.15	7	3.5	
**DASH_l**	Red cabbage	50	22	11	1.5	0.75	0.18	0.09	3.5	1.75	
**DASH_l**	Red bell pepper	50	29	14.5	1.3	0.65	0.6	0.3	4.5	2.25	
**DASH_l**	Kiwi	100	52	52	1.1	1.1	0.5	0.5	10.6	10.6	
**DASH_ma**	Hazelnuts	20	656	131.2	12	2.4	56.25	11.25	10.5	2.1	
**DASH_ma**	Banana	200	89	178	1.2	2.4	0.3	0.6	20	40	
**DASH_d**	Chicken	125	166	207.5	20.85	26.0625	9.25	11.5625	0	0	
**DASH_d**	Tomato	50	19	9.5	0.9	0.45	0.1	0.05	3.5	1.75	
**DASH_d**	Avocado	50	137	68.5	1.5	0.75	12	6	0.4	0.2	
**DASH_d**	Endive	50	12	6	1.6	0.8	0.2	0.1	1	0.5	
**DASH_d**	Orange	200	38	76	0.8	1.6	0	0	8.6	17.2	
**Total**				**1538**		**61.2325**		**57.5225**		**191.66**	
**EEUU_b**	Greek yogurt (plain. low-fat)	200	59	118	10.3	20.6	0.37	0.74	3.64	7.28	2 units
**EEUU_b**	Walnuts	20	730	146	14.6	2.92	69.7	13.94	10.9	2.18	1 handful
**EEUU_b**	Strawberries	150	36	54	0.64	0.96	0.22	0.33	7.96	11.94	1 portion of fruit
**EEUU_b**	Coffee with milk*	200	38	76	1.7	3.4	2.36	4.72	2.45	4.9	1 cup
**EEUU_mm**	Boiled chicken egg	120	143	171.6	245.388	12.3	17.589	10.3	14.729	0.9	2 big units
**EEUU_l**	Pork loin (roasted. lean)	200	168	336	21.1	42.2	9.47	18.94	0	0	
**EEUU_l**	Asparagus (cooked)	150	28	42	11.76	1.44	0.4032	0.22	0.0616	5.1	1ª portion of vegetables
**EEUU_l**	Quinoa (dry)*	30	306	91.8	13.8	4.14	5.56	1.668	49.2	14.76	
**EEUU_l**	Olive oil*	15	899	134.85	0	0	100	15	0	0	1.5 spoons
**EEUU_l**	Banana	120	98	117.6	0.74	0.888	0.29	0.348	23	27.6	2ª portion of fruit
**EEUU_ma**	Cottage cheese	50	103	51.5	11.6	5.8	4.22	2.11	4.6	2.3	

**EEUU_ma**	Almond butter (100%)	15	645	96.75	20.8	3.12	53	7.95	21.1	3.165	
**EEUU_d**	Salmon (baked/grilled)	150	197	295.5	20.3	30.45	13.1	19.65	0	0	
**EEUU_d**	Red bell pepper	100	31	31	0.89	0.89	0.13	0.13	7.68	7.68	
**EEUU_d**	Fresh spinach	100	27	27	2.85	2.85	0.62	0.62	2.41	2.41	
**EEUU_d**	Olive oil*	15	899	134.85	0	0	100	15	0	0	1.5 spoons
**Total**				**1924.45**		**131.958**		**111.666**		**90.215**	
**MD_b**	Whole natural yogurt	250	64	160	3.2	8	3.9	9.75	3.9	9.75	2 units (dairy serving)
**MD_b**	Whole grain oat flakes	60	401	240.6	16.9	10.14	6.9	4.14	66.3	39.78	1 small cup
**MD_b**	Chopped walnuts	20	595	119	14	2.8	63.28	12.656	3.3	0.66	1 handful
**MD_b**	Blueberries or mixed berries	100	33	33	0.63	0.63	0.6	0.6	6.05	6.05	1 small cup
**MD_b**	Herbal tea	100	0	0	0	0	0	0	0	0	Without sugar or milk
**MD_mm**	Apple	150	52	78	0.3	0.45	0	0	12	18	1 medium piece
**MD_mm**	Hazelnuts	20	656	131.2	12	2.4	56.25	11.25	10.5	2.1	1 handful
**MD_l**	Lentils (dry)	70	310	217	24.76	17.332	1.17	0.819	48.69	34.083	1 regular plate
**MD_l**	Onion	50	26	13	1.25	0.625	0	0	5.3	2.65	In the stew
**MD_l**	Carrot	50	34	17	0.8	0.4	0.3	0.15	7	3.5	In the stew
**MD_l**	Extra virgin olive oil	10	888	88.8	0	0	100	10	0	0	1 tablespoon
**MD_l**	Fresh sardines	125	140	175	18.1	22.625	7.5	9.375	0	0	3–4 small units
**MD_l**	Mandarin orange	150	40	60	0.8	1.2	0	0	9	13.5	2 units
**MD_ma**	Fresh cheese (cow's milk)	80	198	158.4	12.4	9.92	15.4	12.32	2.5	2	1 individual portion
**MD_d**	Eggplant	150	20	30	0.7	1.05	0.2	0.3	3.8	5.7	Grilled or pan-seared
**MD_d**	Potato	150	73	109.5	2.2	3.3	0.2	0.3	15.2	22.8	Boiled or baked
**MD_d**	Chicken egg	60	150	90	12.5	7.5	11.1	6.66	0	0	1 large unit
**MD_d**	Extra virgin olive oil	10	888	88.8	0	0	100	10	0	0	1 tablespoon
**MD_d**	Pear	150	45	67.5	0.4	0.6	0	0	10.6	15.9	1 medium piece

**Total**	**1809.3**	**88.972**	**88.32**	**176.473**	

DAY 7

			Calories (kcal)		Proteins (g)		Fats (g)		Carbohydrates (g)		Observations
**Diet**	**Food type**	**Weight (grams)**	**100g**	**Kcal diet**	**100g**	**Grams diet**	**100g**	**Grams diet**	**100g**	**Grams diet**	
**DASH_b**	Whole wheat bread	80	251	200.8	10.9	8.72	3	2.4	44	35.2	
**DASH_b**	Avocado	80	137	109.6	1.5	1.2	12	9.6	0.4	0.32	
**DASH_b**	Unsalted fresh cheese	75	198	148.5	2.5	1.875	15.4	11.55	2.5	1.875	
**DASH_b**	Apricot	150	42	63	0.8	1.2	0	0	9.5	14.25	
**DASH_b**	Herbal tea	200	0	0	0	0	0	0	0	0	
**DASH_b**	Low-fat plain yogurt	125	46	57.5	4.3	5.375	0.32	0.4	6.3	7.875	
**DASH_mm**	Oats	50	401	200.5	16.9	8.45	6.9	3.45	66.3	33.15	
**DASH_mm**	Banana	200	89	178	1.2	2.4	0.3	0.6	20	40	
**DASH_l**	Beans	60	272	163.2	22.23	13.338	1.34	0.804	41.64	24.984	
**DASH_l**	Onion	25	26	6.5	1.25	0.3125	0	0	5.3	1.325	
**DASH_l**	Carrot	75	34	25.5	0.8	0.6	0.3	0.225	7	5.25	
**DASH_l**	Leek	25	24	6	1.6	0.4	0.3	0.075	3.7	0.925	
**DASH_l**	Turnip	25	19	4.75	0.9	0.225	0.2	0.05	3.2	0.8	
**DASH_l**	Pear	150	45	67.5	0.4	0.6	0	0	10.6	15.9	
**DASH_ma**	Hummus	40	360	144	8.9	3.56	2.5	1	18.7	7.48	
**DASH_ma**	Carrot	20	34	6.8	0.8	0.16	0.3	0.06	7	1.4	
**DASH_d**	Eggplant	100	20	20	0.7	0.7	0.2	0.2	3.8	3.8	
**DASH_d**	Quinoa	50	306	153	13.8	6.9	5.56	2.78	49.2	24.6	
**DASH_d**	Red bell pepper	30	29	8.7		0		0		0	
**DASH_d**	Onion	30	26	7.8	1.25	0.375	0	0	5.3	1.59	
**DASH_d**	Zucchini	50	17	8.5	1.8	0.9	0.2	0.1	2	1	
**DASH_d**	Tuna in water	80	101	80.8	23.5	18.8	0.6	0.48	0	0	
**DASH_d**	Kiwi	100	52	52	1.1	1.1	0.5	0.5	10.6	10.6	
**Total**				**1402.55**		**77.1905**		**34.274**		**232.324**	
**EEUU_b**	Chicken egg	120	143	171.6	245.388	12.3	17.589	10.3	14.729	0.9	2 units

**EEUU_b**	Smoked turkey breast*	50	104	52	19.3	9.65	0.8	0.4	3.6	1.8	2-3 slices
**EEUU_b**	Fresh spinach	50	27	13.5	3.645	1.44	0.3888	0.22	0.0594	5.1	1/2 serving of vegetables
**EEUU_b**	Coffee with milk*	200	38	76	1.7	3.4	2.36	4.72	2.45	4.9	1 cup
**EEUU_mm**	Greek yogurt	150	59	88.5	10.3	15.45	0.37	0.555	3.64	5.46	
**EEUU_mm**	Almonds	20	626	125.2	21.4	4.28	51.1	10.22	20	4	1 handful
**EEUU_l**	Beef (tenderloin/lean loin)	200	110	220	20.19	40.38	3.06	6.12	0	0	
**EEUU_l**	Green beans (cooked)	150	40	60	1.97	2.955	0.28	0.42	7.41	11.115	
**EEUU_l**	Sweet potato (baked/roasted)	100	78	78	1.58	1.58	0.38	0.38	17.3	17.3	
**EEUU_l**	Olive oil	15	888	133.2	0	0	100	15	0	0	1.5 tablespoons
**EEUU_l**	Pear	150	63	94.5	0.38	0.57	0.16	0.24	15.1	22.65	
**EEUU_ma**	Cottage cheese	100	103	103	11.6	11.6	4.22	4.22	4.6	4.6	
**EEUU_ma**	Tomato	100	22	22	0.7	0.7	0.42	0.42	3.24	3.24	
**EEUU_d**	Cod (baked)	200	66	132	16.1	32.2	0.67	1.34	0	0	
**EEUU_d**	Carrot (boiled/roasted)	150	48	72	0.94	1.41	0.35	0.525	10.3	15.45	
**EEUU_d**	Avocado	80	223	178.4	1.81	1.448	20.3	16.24	8.32	6.656	1/2 unit (healthy fat)
**EEUU_d**	Olive oil	10	888	88.8	0	0	100	10	0	0	1 tablespoon
**EEUU_d**	Blueberries	100	64	64	0.7	0.7	0.31	0.31	14.6	14.6	
**Total**				**1708.7**		**140.063**		**81.63**		**117.771**	
**MD_b**	Scrambled chicken egg	120	150	180	12.5	15	11.1	13.32	0	0	2 eggs (scrambled)
**MD_b**	Whole wheat bread	60	251	150.6	10.9	6.54	3	1.8	44	26.4	2 slices
**MD_b**	Ripe tomato	50	19	9.5	0.9	0.45	0.1	0.05	3.5	1.75	Slices on top of the bread
**MD_b**	Extra virgin olive oil	10	888	88.8	0	0	100	10	0	0	1 tablespoon
**MD_b**	Coffee with semi-skimmed milk	200	38	76	1.7	3.4	2.36	4.72	2.45	4.9	1 cup
**MD_mm**	Walnuts	20	595	119	14	2.8	63.28	12.656	3.3	0.66	1 handful
**MD_mm**	Apple	150	52	78	0.3	0.45	0	0	12	18	1 medium piece
**MD_l**	Wheat semolina (raw)	80	350	280	12.6	10.08	1.24	0.992	70.4	56.32	1 regular plate

**MD_l**	Shrimp	120	90	108	18	21.6	1.81	2.172	0	0	
**MD_l**	Broccoli	150	26	39	3	4.5	0.4	0.6	2.4	3.6	1 plate of vegetables
**MD_l**	Extra virgin olive oil	10	888	88.8	0	0	100	10	0	0	1 tablespoon
**MD_l**	Orange	200	38	76	0.8	1.6	0	0	8.6	17.2	1 large unit
**MD_ma**	Hummus (chickpeas)	60	360	216	8.9	5.34	2.5	1.5	18.7	11.22	2 tablespoons
**MD_ma**	Carrot	150	34	51	0.8	1.2	0.3	0.45	7	10.5	Cut into sticks (raw)
**MD_d**	Lettuce salad	100	16	16	1.125	1.125	0.6	0.6	1.4	1.4	Base of fresh vegetables
**MD_d**	Ripe tomato	100	19	19	0.9	0.9	0.1	0.1	3.5	3.5	1 mediun piece
**MD_d**	Fresh cheese (cow's milk)	80	198	158.4	2.5	2	15.4	12.32	2.5	2	1 individual portion
**MD_d**	Extra virgin olive oil	10	888	88.8	0	0	100	10	0	0	1 tablespoon
**MD_d**	Whole wheat bread	40	251	100.4	10.9	4.36	3	1.2	44	17.6	1 slice
**MD_d**	Melon	250	27	67.5	0.6	1.5	0	0	6	15	2 slices
**Total**	**Total**			**2010.8**		**82.845**		**82.48**		**190.05**	
